# Elevated Soluble Urokinase Plasminogen Activator Receptor and Proenkephalin Serum Levels Predict the Development of Acute Kidney Injury after Cardiac Surgery

**DOI:** 10.3390/ijms18081662

**Published:** 2017-07-31

**Authors:** Jana C. Mossanen, Jessica Pracht, Tobias U. Jansen, Lukas Buendgens, Christian Stoppe, Andreas Goetzenich, Joachim Struck, Rüdiger Autschbach, Gernot Marx, Frank Tacke

**Affiliations:** 1 Department of Medicine III, University Hospital Aachen, 52074 Aachen, Germany; jmossanen@ukaachen.de (J.C.M.); jessica.pracht@rwth-aachen.de (J.P.); tobias.udo.jansen@rwth-aachen.de (T.U.J.); lbuendgens@ukaachen.de (L.B.); 2 Department of Intensive and Intermediate Care, University Hospital Aachen, 52074 Aachen, Germany; cstoppe@ukaachen.de (C.S.); gmarx@ukaachen.de (G.M.); 3 Department of Thoracic and Cardiovascular Surgery, University Hospital Aachen, 52074 Aachen, Germany; agoetzenich@ukaachen.de (A.G.); rautschbach@ukaachen.de (R.A.); 4 Sphingotec GmbH, 16761 Hennigsdorf, Germany; struck@sphingotec.de

**Keywords:** acute kidney injury, soluble urokinase plasminogen activator receptor, suPAR, proenkephalin, pro-ENK, cardiac surgery, biomarker, acute kidney failure

## Abstract

Acute kidney injury (AKI) develops in up to 40% of patients after cardiac surgery. The soluble urokinase plasminogen activator receptor (suPAR) has been identified as a biomarker for incident chronic kidney disease (CKD). Proenkephalin (proENK) also has been shown to be a biomarker for renal dysfunction. We hypothesized that pre-surgery suPAR and proENK levels might predict AKI in patients undergoing cardiac surgery. Consecutive patients (*n* = 107) undergoing elective cardiac surgery were studied prospectively. Clinical data, laboratory parameters, suPAR and proENK serum levels were assessed before operation, after operation and days one and four post-operatively. A total of 21 (19.6%) patients developed AKI within the first four days after elective surgery. Serum levels of suPAR and proENK, but not of creatinine, were significantly higher before surgery in these patients compared to those patients without AKI. This difference remained significant for suPAR, if patients with or without AKI were matched for risk factors (hypertension, diabetes, CKD). If cardiac surgery patients with pre-existing CKD (*n* = 10) were excluded, only pre-operative suPAR but not proENK serum levels remained significantly elevated in patients with subsequent AKI. Thus, our findings indicate that suPAR may be a predictive biomarker for AKI in the context of cardiac surgery, even in patients without underlying CKD.

## 1. Introduction

Acute kidney injury (AKI) is a well-recognized complication after cardiac surgery with an incidence of up to 40% [1,2,3]. Importantly, even a slight increase of serum creatinine after cardiac surgery (i.e., stage I AKI) is associated with increased mortality [4].

In order to identify subjects at risk and in order to prevent AKI, it is necessary to identify new predictors of renal injury before surgery and during the early post-operative course. Currently, the diagnosis of AKI is primarily based on changes in the serum creatinine concentration, which is an imperfect indicator of renal function and dysfunction. Creatinine concentration is dependent on several clinical variables, such as hydration, nutritional status, muscle metabolism or medication effects, and serum creatinine may not change until a reduction of the glomerular filtration rate (GFR) of around 50% is reached [5,6]. Based on these limitations, creatinine remains an insufficient predictor of AKI, leaving a high demand to develop more accurate biomarkers to predict AKI, particularly for high risk patients undergoing elective cardiac surgery.

Two novel biomarkers with the potential to complement creatinine are the soluble urokinase plasminogen activator receptor (suPAR) and proenkephalin (proENK) A119-159. ProENK has been very recently suggested as a novel biomarker associated with AKI after cardiac surgery [7]. ProENK is a stable surrogate marker for endogenous enkephalins and stems from the same precursor as Met- and Leu-enkephalin [8]. Enkephalins are associated with various physiological functions such as immunity, cardiovascular functions and inflammation [9,10,11], enkephalin and its receptors are highly expressed in renal tissue, suggesting that enkephalin may play an important biological function in renal physiology. Elevated proENK can predict incident kidney diseases in the general population, and an association between proENK levels and the deterioration of kidney function over time has been observed [12,13]. Elevated proenkephalin has also been related to a worse prognosis after acute myocardial infarction [14].

The suPAR molecule is derived from the proteolytic cleavage of the membrane bound urokinase plasminogen activator receptor (uPAR, cluster of differentiation (CD) 87). It is detectable in different body fluids like blood, urine, peritoneal fluids and cerebrospinal fluid [15]. Initially, suPAR was recognized as a potential biomarker of immune activation and inflammation in various disease states [16,17]. As such, suPAR is a predictor for mortality in critically ill patients [18] as well as in cardiovascular disease [19]. More recently, suPAR was pathogenically linked to the development of kidney diseases like focal segmental glomerulosclerosis [20]. In fact, elevated serum suPAR levels were identified as a biomarker predicting incident kidney diseases in large cohorts of healthy volunteers and individuals at risk for chronic kidney disease [21]. Thus, we hypothesized that suPAR levels might be a yet unrecognized biomarker predicting AKI after cardiac surgery.

We therefore conducted a prospective observational trial in 107 consecutive cardiac surgery patients to assess the potential of suPAR and proENK to predict the development of AKI after surgery. We herein demonstrate that elevated suPAR and proENK levels, but not serum creatinine, are associated with subsequent AKI in patients undergoing elective cardiac surgery. The predictive value remained present for suPAR, but not proENK, if patients with pre-existing chronic kidney diseases were excluded from the analysis, suggesting a novel role of suPAR as a biomarker for predicting post-surgery AKI in cardiovascular patients.

## 2. Results

### 2.1. Patient Characteristics and Incidence of Acute Kidney Injury

The baseline clinical characteristics of the 107 patients participating in our study are displayed in Table 1. As expected for a cohort undergoing elective cardiac surgery, patients had typical co-morbidities and medications. Among the 107 patients, 21 (19.6%) developed AKI at a median of one day (range 1–4 days) after surgery. In the majority, AKI was stage one (17/21, 81%), while AKI stage two (3/21, 14.3%) and stage three (1/21, 4.7%) were rare. Patients developing AKI had a higher prevalence of chronic kidney disease, more frequent use of diuretics—potentially indicating a reduced renal reserve [22,23,24]—and higher white blood cells counts at baseline. Moreover, they more often underwent dual bypass and valve replacement surgery (Table 1). Since the total surgery time was not increased in patients with subsequent AKI, the dual surgery might be an indicator of additional risk factors such as comorbidities or age.

After surgery, participants developing AKI within the first four days stayed longer at the intensive care unit (ICU), and simplified acute physiology score (SAPS) at day one and sequential organ failure assessment (SOFA) scores at days one and four were higher compared to patients without AKI. There were no significant differences between patients with AKI and without AKI with respect to the duration of myocardial ischemia or co-medications like angiotensin-converting-enzyme (ACE) inhibitors, angiotensin II (ATII) receptor antagonists, statins and calcium channel blockers (Table 1). The overall incidence of AKI in our cohort (20%) differs from some studies in patients undergoing cardiac surgery that reported up to 40%; however, the age and ischemia time of the patients from our cohort appears more favorable than in prior studies (Table 1) [25,26].

### 2.2. suPAR and proENK in Patients Developing Acute Kidney Injury after Cardiac Surgery

**Figure 1:**
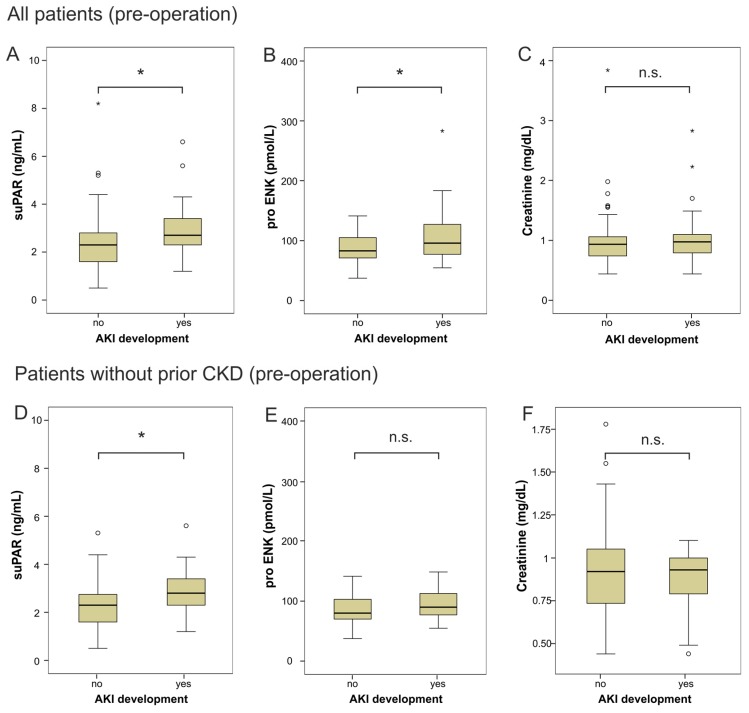

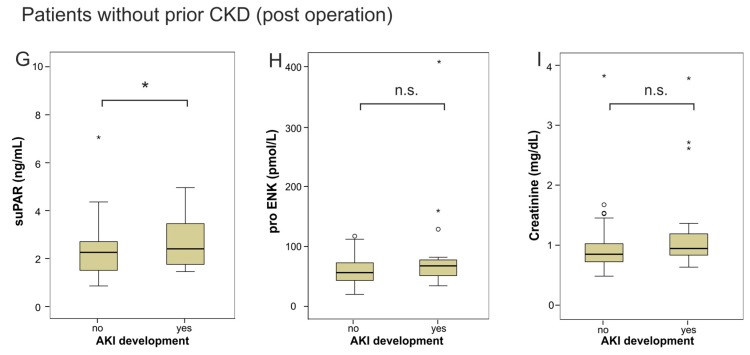
Serum concentrations of different biomarkers for AKI before surgery. (**A**) Serum suPAR levels were determined by enzyme linked immunoassay (ELISA) and revealed significantly higher levels at baseline for patients developing AKI within four days after surgery than patients without AKI. (**B**) Serum proENK levels were significantly elevated in patients with AKI after surgery. (**C**) In contrast, serum creatinine levels were not different between the two groups. (**D**–**F**) Serum levels of suPAR, proENK and creatinine of patients before cardiac surgery, excluding patients with the diagnosis of chronic kidney disease (CKD) such as diabetic nephropathy. (**G**–**I**) Serum levels of suPAR, proENK and creatinine immediately after cardiac surgery, excluding patients with chronic kidney disease. Box plot are displayed, where the bold line indicates the median per group, the box represents 50% of the values. The horizontal lines show minimum and maximum values of calculated non-outlier values; asterisks and open circles indicate outlier values (* *p* < 0.05, ** *p* < 0.01).

Serum levels of suPAR, proENK and creatinine were analyzed before operation and at admission to the ICU immediately after cardiac surgery. Interestingly, the pre-operative suPAR and proENK levels were significantly elevated in patients developing AKI after cardiac surgery compared to patients without AKI (suPAR median 2.8 ng·mL^−1^, range 1.2–6.6, vs. 2.3, 0.5–240, *p* = 0.021; pro ENK median 96 pmol·L^−1^, range 55–587, vs. 84, 37–1157, *p* = 0.037; Figure 1A,B). In contrast, serum creatinine levels did not differ between these groups (Figure 1C).

Ten patients (9%) of the total cohort had been diagnosed with chronic kidney disease (CKD) before and might therefore be exceptionally susceptible to AKI. SuPAR and proENK, were significantly elevated at baseline (prior to surgery) in CKD patients. The median suPAR for CKD was 2.8 ng·mL^−1^ (*n* = 10) vs. 2.3 ng·mL^−1^ (*n* = 97, *p* = 0.027), the median proENK for CKD was 132.5 pmol·L^−1^ (*n* = 10) compared to 81.7 pmol·L^−1^ (*n* = 97, *p* < 0.001) in patients without CKD. When we excluded patients with preexisting CKD, suPAR concentrations at baseline remained elevated in patients developing AKI within the first four days after cardiac surgery (median 2.9 ng·mL^−1^, range 1.2–5.6, vs. 2.3, 0.5–240, *p* = 0.016) (Figure 1D). In contrast, proENK levels and serum creatinine levels did not differ amongst those patients who did or did not develop AKI (Figure 1E,F). We next tested whether the levels of these biomarkers directly after surgery would indicate subsequent AKI. In all patients as well as in patients without prior CKD (Figure 1G), serum levels of suPAR were significantly elevated post-operation in patients with subsequent AKI development (median 3.1 ng·mL^−1^, range 1.4–4.9, vs. 2.2, 0.8–135, *p* = 0.007). This was not observed for post-surgery proENK (Figure 1H) or creatinine (Figure 1I) levels.

### 2.3. Risk Prediction for AKI by suPAR and proENK

**Figure 2:**
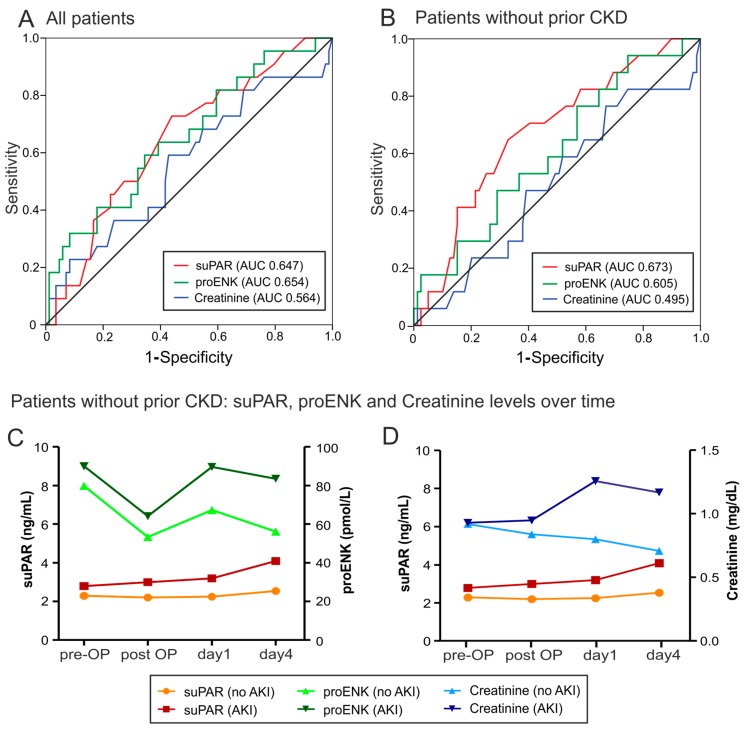
Predictive value of AKI biomarkers and longitudinal assessment. (**A**) and (**B**) receiver operating characteristic (ROC) curve analyses for pre-operative suPAR, proENK and creatinine to predict post-surgery AKI for all patients (**A**) and patients without prior CKD (**B**). (**C**) suPAR and proENK serum levels over time (pre- and post-operation, day 1 and day 4 after operation) for patients without prior CKD. (**D**) SuPAR and creatinine serum levels over time in patients developing AKI compared to patients without AKI development. AUC: Area under the curve.

Based on pre-operative elevated serum levels of suPAR and proENK in patients developing AKI, we hypothesized that suPAR and proENK measurements could predict AKI development after cardiac surgery. We therefore compared suPAR and proENK serum concentrations before operation in patients developing AKI to those who remain without AKI by receiver operating characteristic (ROC) curve analysis. Specifically, elevated suPAR levels before cardiac surgery were a prognostic predictor for AKI development (Figure 2A). The value of suPAR for predicting AKI was even higher, if we excluded patients with CKD (Figure 2B). By univariate regression analysis, pre-existing CKD (*p* = 0.016), leukocyte count (*p* = 0.036), use of diuretics (*p* = 0.004), suPAR > 2.45 ng·mL^−1^ (*p* = 0.018), and proENK > 93.2 pmol·L^−1^ (*p* = 0.047) were predictors of AKI, whereas the patient’s age, operation time or extracorporal circulation time were not associated with the development of AKI (Table 1). However, the number of events (=number of patients developing AKI) was too low to allow sufficient multivariable regression analyses for these predictive factors (suPAR, proENK, CKD). The detailed analysis of the diagnostic accuracy of suPAR and proENK for the identified cut-off values is shown in Table 2.

In order to adjust the predictive value of suPAR and proENK for AKI to potential confounders, we matched patients with AKI (*n* = 18) to a cohort of non-AKI patients (*n* = 18) with the same risk profile for AKI (hypertension, CKD and diabetes). In this analysis, suPAR serum levels prior to surgery were still significantly elevated in patients developing AKI (suPAR median 2.9 ng·mL^−1^, range 1.2–5.6 vs. 2.3, 0.5–8.2, *p* = 0.029). ProENK did not retain its statistically significant difference between the two groups matched for AKI risk factors.

Interestingly, the three potential biomarkers of kidney failure (suPAR, proENK and creatinine) showed strikingly different kinetics during longitudinal measurements. SuPAR levels increased in patients developing AKI, and this increase continued till day four, when creatinine levels no longer increased (Figure 2C,D). ProENK levels dropped immediately after surgery in patients irrespective of subsequent AKI (Figure 2C). Creatinine levels showed a modest increase post-surgery in patients developing AKI (Figure 2D).

## 3. Discussion

In this study, we assessed suPAR and proENK as potential new biomarkers for AKI in patients undergoing cardiac surgery. In these patients, suPAR serum concentrations before and after operation were found to have a close association with the incidence of AKI within the first four days after surgery.

By direct comparison, the predictive value of suPAR appears to be higher if patients with pre-existing CKD as a prominent risk factor for AKI [1] are excluded. A recent similar study with 92 patients (20 developed AKI) reported a correlation between pre-operative proENK serum concentrations and AKI development after cardiac surgery [7]. The absolute values of proENK were very similar to our study as well as the reported predictive value of proENK for AKI in cardiac surgery patients (AUC 0.683 compared to AUC 0.651 in our study) [7]. However, when we excluded patients with pre-existing CKD, a subgroup analysis not conducted in the comparable study [7], the pre-operative serum levels of proENK were not significantly elevated in our patients developing AKI after the operation. Nonetheless, only a small fraction of our cohort had CKD at baseline, emphasizing the need for larger prospective analyses. The observed drop of proENK immediately after the surgery might be due to the induced cardiac arrest, since the heart has also been described as a source for encephalin expression [13]. It has been suggested that proENK is a good biomarker for AKI due to reduced filtration and reabsorption in the injured kidney as well as upregulation based on kidney injury [27]. Large multicenter prospective trials are required to answer the question whether proENK is a useful biomarker for AKI in the context of cardiac surgery or if it is only elevated due to the high proportion of patients with preexisting CKD that develop AKI after operation.

More interestingly, we show for the first time the predictive value of suPAR for AKI after cardiac surgery. Serum suPAR levels were higher in patients developing AKI both pre-operation and post-operation. Importantly, these findings remained significant both during the inclusion and exclusion of patients with prior CKD. suPAR has several advantageous properties as a potential biomarker due to its high stability in serum samples and limited circadian changes in plasma concentrations [28]. We speculate that suPAR > 2.45, which is the result of uPAR shedding from leukocytes, might reflect a low-grade inflammation that is a risk factor for AKI. In agreement, white blood cells were correlated with suPAR (*r* = 0.235, *p* = 0.016) and also associated with AKI development in our cohort.

Slightly higher numbers of patients developing AKI underwent coronary artery bypass (CABG) plus aortic valve replacement (AVR), while the single surgeries (CABG or AVR only) did not differ between both groups. Based on comparable surgery or CBP times between the AKI and non-AKI group, the slight imbalance is possibly an indicator of more medical comorbidities in patients developing AKI, likewise reflected in the higher diuretic use in this group.

Establishing a reliable cut-off value for suPAR appears challenging, though, since suPAR levels in AKI patients in this study were essentially within the reported healthy normal range (<4 ng·mL^−1^). However, it has been previously noted in large prospective cohorts that suPAR levels of the third and fourth quartile of a “normal” population can be predictive for kidney failure [21]. We have demonstrated before that suPAR levels are associated with renal function in patients with critical illness and sepsis [16] as well as in patients with compensated and decompensated liver cirrhosis [13,29]. Recently Hayek at al. showed that elevated plasma suPAR levels are independently associated with the incidence of CKD and an accelerated decline in the glomerular filtration rate (GFR) in volunteers with a cardiovascular risk profile, independent of conventional risk factors for cardiovascular disease and CKD [21]. Previous studies have indicated that suPAR has a pathological role in kidney diseases like focal segmental glomerulosclerosis, diabetic nephropathy and lupus nephritis [20,30,31]. To validate the influence of the GFR, glomerular and tubular damage or inflammation on serum levels of suPAR and proENK, the exact clinicopathological correlation remains to be investigated. Our study provides a potential practical application of these observations to clinical algorithms, as elevated baseline suPAR in the context of cardiovascular disease was indicative of subsequent AKI after elective cardiac surgery. Thus, suPAR levels could potentially help in the identification and risk stratification of this patient population. However, the best cut-off for suPAR (>2.45 ng·mL^−1^ in our study) as well as the optimal pre-, intra- and post-surgical management requires further investigation. It is possible that suPAR added to combination of known AKI biomarkers including interleukin-18 or neutrophil gelatinase-associated lipocalin (NGAL) can strengthen the prediction of those at risk for the development and progression of AKI [27,32].

### Limitations of Our Study

Our study was a single center study with a limited number of patients. Thus, the number of events, especially advanced stage AKI, was too low for extensive subgroup analyses or extensive multivariable regression analyses. Moreover, the assessment of AKI was based on creatinine increase (KDIGO criteria) without extensive additional analyses (e.g., urine sedimentation, renal histology). In addition, the long-term consequences of post-surgery AKI also warrant further analyses, as our study was designed to assess 90-day mortality but not longer follow-up periods or different endpoints. It can be anticipated that larger prospective multicenter studies may allow us to better define the predictive value of both biomarkers for AKI in this group of patients.

## 4. Methods

### 4.1. Study Design and Patient Characteristics

A total of 107 patients (77 male, 30 female) were consecutively enrolled in this prospective, observational study after the approval of the local institutional review board and informed consent. Clinical Trials.gov identifier: NCT02488876. All patients provided written informed consent. The local ethics’ committee approved this study under (151/09).

All patients that presented for elective cardiac surgery were included over a pre-specified time period from 1 June 2014 to 31 November 2015 on one pre-defined day of the week. Chronic kidney disease (CKD) was pre-operatively assessed in a standardized manner (medical records, laboratory analyses); two patients were receiving chronic hemodialysis due to stage V CKD. Exclusion criteria were known or suspected pregnancy, emergency operations and patients aged less than 18 years. Patient data, clinical information and blood samples were collected prospectively immediately before (pre) surgery, after (post) surgery as well as one day and four days after cardiac surgery. Serum samples were available before surgery in 107/107, directly after surgery in 93/107, at day one in 95/107 and at day four in 70/107 patients.

The clinical course of the patients was observed in a follow-up period by directly contacting the patients, the patients’ relatives or their primary care physician. Patients fulfilling the criteria of AKI by the KDIGO criteria within the first four days after surgery were categorized as patients with AKI development, the others as patients with no AKI development [33].

### 4.2. Management of Anesthesia and Surgical Procedures

Anesthesia was performed according to our institutional routine. Fluid substitution was performed with 1 mL·kg^−1^·h^−1^ balanced crystalloid solutions. After surgery, all patients were transferred to the ICU, and the following post-operative treatment was standardized according to our institutional guidelines.

The surgical procedure with use of conventional cardiopulmonary bypass (CPB, “on-pump”) was performed in accordance to our clinical standards. The extracorporeal circulation was performed with a non-pulsatile pump flow of 2.2 L·min^−1^·m^2^, targeting a blood pressure between 50 and 70 mmHg. Cardiac arrest was introduced by a single antegrade infusion of cold crystalloid cardioplegic solution into the aortic root immediately after crossclamping. After CPB, heparin was antagonized with protamine in a ratio of 1:1, and aspirin was administered orally starting at 8 h post-operatively. The off-pump technique was performed as described before [34].

### 4.3. Determination of suPAR, proENK and Creatinine Serum Concentrations

All blood samples were immediately placed on ice and, after centrifugation, stored at −80 °C. Serum levels of suPAR were measured using the suPARnostic enzyme linked immunoassay (ELISA) (ViroGates, Birkerød, Denmark). ProENK levels were measured by immunoluminometric assay (Sphingotec GmbH, Hennigsdorf, Germany), as described before [7]. Creatinine measurements were done by clinical routine determinations. Baseline creatinine was based on laboratory tests within five days prior to surgery. All samples were measured in a blind fashion, in which the technicians were unaware of the group attribution or other clinical or laboratory data.

### 4.4. Statistical Analysis

All statistical analyses were performed using GraphPad Prism 5.0 (Graphpad Software Inc., San Diego, CA, USA) and SPSS 23 (SPSS Inc., Chicago, IL, USA), as described before [35]. Patient data are displayed as median and range providing the skewed distribution of most parameters. After testing for normal distribution (Shapiro–Wilk W-test), differences between groups were tested using corresponding analysis of variance tests. Post hoc testing was performed using Bonferroni adjustments for multiple measurements. For non-normal distributed data, the Mann–Whitney U-test was used to calculate the differences between the two groups, and multiple comparisons between more than two groups were performed by Kruskal–Wallis analysis of variance (ANOVA) and the Mann–Whitney U-test for post hoc analysis.

Box plot graphics illustrate comparisons between subgroups and they display a statistical summary of the median, quartiles, range and extreme values. The whiskers extend from the minimum to the maximum value excluding outside and far out values which are displayed as separate points. An outside value (indicated by an open circle) was defined as a value that is smaller than the lower quartile minus 1.5 times the interquartile range, or larger than the upper quartile plus 1.5 times the interquartile range. A far outlying value was defined to be smaller than the lower quartile minus three times the interquartile range or larger than the upper quartile plus three times the interquartile range. All values have been included for statistical analyses. The prognostic value of the variables was tested by univariate and multivariable analysis in the linear regression model [36]. Receiver operating characteristic curves were generated by plotting sensitivity against 1-specificity. Values presenting serum levels over time are displayed as the summary of median values.

## 5. Conclusions

Our study identifies suPAR and proENK as potential biomarkers to predict the development of AKI in cardiovascular disease patients undergoing elective cardiac surgery. Both markers were significantly elevated at baseline in patients developing AKI. While the predictive value of suPAR even rises if patients with preexisting chronic kidney disease are excluded from the analysis, proENK loses its predictive power in this subgroup analysis. These data support the hypothesis that especially suPAR is a promising novel biomarker for AKI in the context of cardiac surgery. Therefore, studies confirming this notion with larger patient cohorts in a multicenter setting are warranted to develop new strategies involving this biomarker in individual risk prediction and AKI prevention after elective heart surgery.

## Figures and Tables

**Table 1:** Baseline patient characteristics.

Parameter	All Patients	Non-AKI	AKI	*p*-Value
	*n* = 107	*n* = 86	*n* = 21	
**Demographics**				
Sex (male/female)	77/30	62/24	15/6	0.952
Age median (IQR) (years)	69 (61–76)	67 (61–75)	75 (66–79)	0.062
Body mass index (IQR) (BMI)	27 (24–30)	27 (24–30)	27 (25–30)	0.613
30 days Mortality *n* (%)	1 (1)	0 (0)	1 (5)	0.186
90 days Mortality *n* (%)	3 (3)	1 (1)	2 (10)	0.110
**Surgery and ICU observation**				
CABG *n* (%)	54 (51)	47 (55)	7 (32)	0.080
CABG + AVR *n* (%)	17 (16)	10 (12)	7 (32)	0.015
AVR *n* (%)	9 (8)	6 (7)	3 (14)	0.279
Bentall, David or Hemashield *n* (%)	8 (7)	7 (8)	1 (5)	0.598
Other cardiac surgery *n* (%)	28 (26)	16 (19)	3 (13)	0.642
Ischemia time (IQR) (min)	79 (60–110)	79 (60–109)	83 (60–114)	0.750
Time of CBP (IQR) (min)	125 (101–156)	125 (100–160)	124 (101–156)	0.729
Total time of surgery (min)	251 (220–290)	252 (217–287)	249 (220–315)	0.476
**Postoperative period**				
ICU days median (IQR) (days)	3 (1–12)	3 (1–10)	5 (2–12)	<0.001
SAPS day1 median (IQR)	29 (26–35)	29 (25–33)	35 (28–39)	0.027
SAPS day4 median (IQR)	24 (19–31)	22 (17–28)	29 (21–37)	0.060
SOFA day1 median (IQR)	5 (3–7)	5 (3–6)	6 (5–7)	0.011
SOFA day4 median (IQR)	0 (0–3)	0 (0–2)	3 (1–5)	<0.001
Nephrotoxic antibiotics *n* (%)	3 (3)	2 (2)	1 (5)	0.484
Dialysis *n* (%)	3 (3)	2 (2)	1 (5)	0.484
**AKI stages**				
Stage I *n* (%)	17 (16)	0 (0)	17 (81)	
Stage II *n* (%)	3 (3)	0 (0)	3 (14)	
Stage III *n* (%)	1 (1)	0 (0)	1 (5)	
**Comorbidities**				
Diabetes *n* (%)	39 (36)	32 (37)	7 (33)	0.741
Hypertension *n* (%)	81 (76)	64 (74)	17 (81)	0.531
Chronic kidney disease *n* (%)	10 (9)	5 (6)	5 (23)	0.011
**Medication**				
Diuretics use *n* (%)	54 (50)	37 (43)	17 (81)	0.002
β–blocker use *n* (%)	75 (70)	61 (71)	14 (67)	0.702
AT II receptor antagonist use *n* (%)	24 (22)	16 (19)	8 (36)	0.055
ACE inhibitor use *n* (%)	52 (49)	43 (50)	9 (43)	0.557
Statin use *n* (%)	67 (63)	53 (62)	14 (64)	0.669
Calcium channel blocker use *n* (%)	24 (22)	16 (9)	8 (36)	0.055
**Laboratory parameters**				
WBC median (IQR) (×10^3^ µL^−1^)	7.9 (6.4–10.5)	7.5 (6.2–9.9)	9.0 (8.2–13.3)	0.009
Creatinine pre-OP (IQR) (mg·dL^−1^)	1 (0.74–1.1)	0.9 (0.74–1.1)	1 (0.7–1.1)	0.269
eGFR pre-OP (IQR) (mL·min^−1^)	75 (63–90)	77 (65–90)	70 (48–90)	0.233
suPAR pre-OP (IQR) (ng·mL^−1^)	2.4 (1.7–3.1)	2.3 (1.6–2.8)	2.8 (2.3–3.4)	0.021
proENK pre-OP (IQR) (pmol·L^−1^)	85 (72–111)	84 (71–105)	96 (77–127)	0.037

AKI: Acute kidney injury; ACE: Angiotensin-converting-enzyme; AT: Angiotensin; AVR: Aortic valve repair; CABG: Coronary artery bypass graft; CBP: Cardiopulmonary bypass; eGFR: Estimated glomerual filtration rate; ICU: Intensive care unit; IQR: Interquartile range; OP: Operation; proENK: Proenkephaline; SAPS: Simplified acute physiology score; SOFA: Sequential organ failure assessment; suPAR: Soluble urokinase plasminogen activator receptor; WBC: White blood cell count.

**Table 2:** Diagnostic accuracy of pre-surgery suPAR and proENK, using optimal cut-off values, for predicting AKI.

	Cut-Off	Sensitivity	Specificity	Youden Index	LHR+	LHR−	Diagnostic Odds Ratio
suPAR	2.45	0.73	0.57	0.30	1.70	0.47	3.58
proENK	93.2	0.59	0.65	0.24	1.69	0.63	2.67

LHR+: Positive likelihood ratio; LHR−: Negative likelihood ratio.
